# Identification of PET/CT radiomic signature for classification of locally recurrent rectal cancer: A network-based feature selection approach

**DOI:** 10.1016/j.heliyon.2024.e41404

**Published:** 2024-12-20

**Authors:** Sara Dalmonte, Maria Adriana Cocozza, Dajana Cuicchi, Daniel Remondini, Lorenzo Faggioni, Paolo Castellucci, Andrea Farolfi, Emilia Fortunati, Alberta Cappelli, Riccardo Biondi, Arrigo Cattabriga, Gilberto Poggioli, Stefano Fanti, Gastone Castellani, Francesca Coppola, Nico Curti

**Affiliations:** a IRCCS Rizzoli Orthopedic Institute, Medical Technology Laboratory, Bologna, 40138, Italy; b Medical Physics Specialization School, University of Bologna, Bologna, 40127, Italy; c Department of Radiology, IRCCS Azienda Ospedaliera-Universitaria di Bologna, Bologna, 40138, Italy; d Medical and Surgical Department of Digestive, Hepatic and Endocrine-Metabolic Diseases, IRCCS Azienda Ospedaliera-Universitaria di Bologna, Bologna, 40138, Italy; e Department of Physics and Astronomy, University of Bologna, Bologna, 40127, Italy; f Nuclear Medicine, IRCCS Azienda Ospedaliero-Universitaria di Bologna, Bologna, 40138, Italy; g IRCCS Institute of Neurological Sciences of Bologna, Data Science and Bioinformatics Laboratory, Bologna, 40139, Italy; h Department of Medical and Surgical Sciences, University of Bologna, Bologna, 40138, Italy; i Dipartimento Diagnostica per Immagini AUSL Romagna, UOC Radiologia Faenza, Faenza, 48018, Italy; j Academic Radiology, Department of Translational Research and of New Surgical and Medical Technologies, University of Pisa, Pisa, 56126, Italy

**Keywords:** Rectal cancer, Recurrence, Machine learning, Radiomics, PET, CT, Medical imaging

## Abstract

**Background:**

The modern approach to treating rectal cancer, which involves total mesorectal excision directed by imaging assessments, has significantly enhanced patient outcomes. However, locally recurrent rectal cancer (LRRC) continues to be a significant clinical issue. Identifying LRRC through imaging is complex, due to the mismatch between fibrosis and inflammatory pelvic tissue. This work aimed to develop a machine learning model for predicting LRRC using radiomic features extracted from 18F-FDG Positron Emission Tomography/Computed Tomography (PET/CT).

**Methods:**

CT and PET images of PET/CT examinations were retrospectively collected from 44 patients, with 29 cases of recurrence (66 %) and 15 cases with no local recurrence (34 %). The whole analysis was conducted separately for CT and PET images to evaluate their different predictive power. Radiomic features were extracted from suspected lesion volumes identified by physicians and the most relevant radiomic features were selected to predict the presence or absence of LRRC. Feature selection was performed using a novel approach derived from gene expression analysis, based on the DNetPRO algorithm. The prediction was done using a Support Vector Classifier (SVC) with a 10-fold cross-validation. The efficiency of the pipeline in predicting LRRC was evaluated according to the sensitivity, specificity, Balanced Accuracy Score (BAS) and Matthews's Correlation Coefficient (MCC).

**Results:**

CT features were found to be the most predictive, showing a sensitivity of 0.80, a specificity of 0.82, a BAS of 0.81 and an MCC of 0.61. PET features obtained a sensitivity of 0.93, a specificity of 0.61, a BAS of 0.77 and a MCC of 0.52. The combination of PET and CT features obtained a sensitivity of 0.80, a specificity of 0.75, a BAS of 0.77 and a MCC of 0.53.

**Conclusions:**

To the best of our knowledge, the DNetPRO algorithm was applied for the first time to medical image analysis and proved suitable for the selection of radiomic features with the highest predictive power, a crucial step in a radiomic pipeline. Our results confirmed the efficiency of radiomic features in predicting LRRC, with CT features outperforming PET features in identifying the characteristic texture of LRRC. The combination of both yielded no performance improvement.

## Highlights

• The proposed pipeline provides a fully automated solution for patient stratification.
• The proposed pipeline performs a robust feature selection, providing hints about novel approaches in radiomic analysis.
• We prove the importance of CT-based radiomic features by comparing their performance with PET-based ones.
• We discussed the possible interpretation of the radiomic features identified from a medical point of view.

## 1 Introduction

The treatment of rectal cancer and its recurrence is based on a multidisciplinary approach, whereby expertise from multiple specialized teams is essential. Combined therapeutic methods have greatly enhanced the treatment results for locally advanced rectal cancer. The effective use and fine-tuning of total mesorectal excision (TME), along with the consistent application of neoadjuvant chemoradiotherapy (nCRT), has substantially lowered local recurrence rates after surgery from 20–30 % to 5–10 % [1]. Despite these advances, locally recurrent rectal cancer (LRRC) continues to be a significant medical challenge.

If LRRC is not treated, it generally advances, infiltrating nearby pelvic organs and nerves, leading to severe symptoms including persistent pelvic pain, bleeding, unpleasant rectal or vaginal discharge, and bowel complications [2]. Without treatment or with palliative care alone, LRRC is typically fatal within 3–12 months [3].

Treatment with a curative aim is the only real chance to save and maintain a patient's quality of life. When carried out at specialized institutions, radical resection has shown a potential 5-year survival rate of 45–50 % [4]. Early LRRC diagnosis undeniably improves the odds of successful treatment.

The diagnosis of rectal cancer recurrence is a complex process involving a combination of clinical examinations, imaging studies, and laboratory tests. Timely and regular follow-up post initial treatment, coupled with advances in diagnostic modalities, plays a pivotal role in the early detection and management of LRRC. Factors suggesting a higher risk of LRRC include postoperative CEA levels, surgical aspects (higher residual tumor [R] classification, with R1/2 being worse than R0, intraoperative perforation, abdominoperineal resection), and tumor characteristics such as advanced T-stage, low rectal tumors, positive circumferential resection margin (CRM), lymphovascular invasion, nodal metastasis, extramural venous invasion and poor tumor differentiation [3,5,6].

Diagnostic modalities face the significant challenge of distinguishing between fibrosis, inflammatory tissue, and tumor growth in areas that have been altered by surgery and radiation. According to clinical guidelines, computed tomography (CT) is the recommended imaging modality for detecting LRRC [[7], [8], [9]]. However, while a growing pelvic lesion observed in consecutive post-operative CT examinations is highly suggestive of LRRC, early detection remains difficult. The overlap between post-surgical changes, such as fibrosis, and tumor regrowth complicates the accuracy of CT imaging alone.

Advanced imaging techniques, such as positron emission tomography/computed tomography with [18F]fluorodeoxyglucose (18F-FDG PET/CT) and pelvic magnetic resonance imaging (MRI), are often used to further investigate suspicious findings from CT studies. MRI is primarily used for surgical planning, but its role in accurately detecting LRRC is still unclear. T1-and T2-weighted MRI sequences, along with contrast-enhanced images, can help identify features of malignancy [10]. Diffusion-weighted imaging (DWI) can be useful in detecting recurrences that might initially be missed, as fibrosis generally presents with low signal intensity, whereas residual tumor tissue exhibits higher signal intensity [11].

Also PET/CT has important limitations. One of the primary challenges is that benign inflammation can sometimes mimic LRRC in PET/CT scans. Recurrence typically shows higher uptake of 18F-FDG compared to fibrosis or fat necrosis, due to the increased glucose metabolism associated with tumor growth [12,13]. However, 18F-FDG uptake is not only driven by glucose metabolism but also by cellular density, which can lead to diagnostic pitfalls. Tumors with low cellularity, such as mucinous and signet-ring cell carcinomas, may result in false negatives due to their low 18F-FDG uptake [14,15]. Conversely, active inflammation can produce false positives due to increased 18F-FDG absorption.

When compared to MRI, 18F-FDG PET/MRI has been shown to improve reader confidence and reduce ambiguous results, even though the diagnostic accuracy of the two modalities is similar [16]. Despite the value provided by these imaging techniques, definitive diagnosis still often requires histopathological confirmation through biopsy, which can delay treatment decisions and sometimes necessitate multiple biopsies.

In view of the limitations of current imaging techniques, particularly in distinguishing between benign post-treatment changes and true tumor recurrence, this study aims to explore alternative approaches or combinations of diagnostic tools to improve the accuracy and timeliness of LRRC detection. In recent years, radiomics has become an emerging research method for extracting, from routinely acquired medical images, thousands of quantitative features not easily perceptible to the radiologist's eye that can be used to build machine learning (ML) models to support diagnostic, prognostic, and predictive clinical decisions [[17], [18], [19], [20]].

Several authors have proposed radiomic solutions for the assessment of colorectal cancer with different purposes [[21], [22], [23]], including prediction of metastases [1,24,25], prediction of response to chemotherapy (neoadjuvant) treatments [[26], [27], [28], [29]], detection of tumor phenotypes [30] and prediction of survival [[31], [32], [33], [34]]. However, few studies have addressed the application of radiomics to the prediction of LRRC, which is still a challenging clinical problem [35,36]. Chen et al. [37] developed an MRI-based radiomic model to assess the presence of LRRC, showing that combining multiple sequences significantly improves model performance. Xie et al. [38] developed and validated a CT radiomics-based nomogram with good predictive performance in LRRC, enhancing early intervention and outcomes. Badic et al. [39] built a model incorporating radiomic CT features and clinical variables with good predictive performance in LRRC, which could help stratify patients after surgery and adapt the treatment approach. Cuicchi et al. [40] showed that the analysis of radiomic features extracted from CT and PET images can be useful in detecting LRRC non-invasively, emphasizing that the features with the highest information power are related to the high local heterogeneity of the tissue.

The combination of radiomics and ML methods undoubtedly holds promise in enhancing our understanding of LRRC. While radiomics offers the opportunity to take full advantage of the enormous amount of data contained in medical images, ML enables classification tasks that obviate the problems of operator subjectivity and possible human error and speed up decision making. As the field evolves, there is hope that the proposed approach will play a crucial role in revolutionizing rectal cancer prognosis and treatment, by improving the earliness of diagnosis and reducing invasiveness for the patient. Despite significant advancements in the diagnosis and treatment of LRRC, a notable gap remains in the literature regarding the application of radiomics for LRRC prediction. While numerous studies have explored the use of radiomics in other aspects of colorectal cancer, such as predicting metastases and treatment responses, few have specifically addressed non-invasive prediction of LRRC. This study seeks to fill this gap by developing a binary classification model using radiomic features extracted from PET/CT images. The goal is to enhance early diagnosis and reduce the reliance on invasive procedures, thereby offering a meaningful contribution to the field and potentially revolutionizing the clinical management of LRRC.

To select PET/CT radiomic signatures among all features extracted from the volumes of suspected lesions identified by experienced physicians, a new method was applied based on the DNetPRO network-based feature selection algorithm [41]. Finally, a Support Vector Classifier (SVC), a standard tool in ML applications [42,43], was used for LRRC prediction. PET/CT images of 44 patients treated at IRCCS Policlinico Sant’Orsola-Malpighi between January 2007 and May 2021 were retrospectively selected for the study.

The highlights of the present study are the following.

• We propose a novel radiomic feature selection method based on the DNetPRO network-based algorithm.
• Heterogeneous texture of rectal tissue undergoing local recurrence is detected by CT radiomic features.
• CT radiomics proved to be a better LRRC predictor than PET radiomics.
• Combining features from PET and CT images do not improve LRRC prediction performance.

## 2 Materials and method

### 2.1 Study population and patient selection

The study was conducted in accordance with the Declaration of Helsinki and approved by the Institutional Review Board (or Ethics Committee) of IRCCS University Hospital of Bologna (protocol code n°. 848/2020/OSS/AOUBo).

Patients who underwent radical resection (R0, with more than a 1 mm margin) of primary rectal adenocarcinoma, with or without preliminary neoadjuvant therapy, between January 2007 and May 2021 at the IRCCS Azienda Ospedaliero-Universitaria di Bologna Division of Colorectal Surgery were evaluated for potential inclusion in this study. Inclusion criteria were the following: i) verified histological LRRC; ii) LRRC suspected through imaging, but not confirmed histologically; iii) at least one follow-up PET/CT suggesting LRRC accessible on the local PACS. Patients were excluded if they had: i) undergone palliative primary tumor resection; ii) R1 primary tumor resection (marginal microscopic resection of 1 mm); iii) R2 primary tumor resection (resection margins visibly affected by the tumor); iv) local excision of primary tumor; v) mucinous adenocarcinoma; vi) lacked histological LRRC confirmation and had less than 18 months of monitoring; vii) unavailable or corrupted image data on the local PACS.

It is important to highlight that mucinous adenocarcinomas often exhibit distinct biological and imaging characteristics compared to other tumor types. Including these cases could have significantly impacted the performance and generalizability of the radiomic models developed in this study, potentially limiting the broader applicability of the conclusions.

Each patient's treatment plan was discussed by the Multidisciplinary Rectal Cancer Team at the IRCCS Azienda Ospedaliero-Universitaria di Bologna and treatment choices for the primary tumor (whether preliminary neoadjuvant therapy or upfront surgery), were based on the clinical stage and tumor location.

Follow-up was conducted every six months for the first five years post-surgery. This involved physical examinations, digital rectal examinations, serum tumor marker tests for Carcinoembryonic Antigen (CEA), endorectal ultrasound when feasible, and yearly chest and abdominopelvic CT imaging. Colonoscopies were scheduled at 6 months post-surgery, at the 2-year and 5-year marks, and every 3–5 years thereafter. If there was any clinical indication of LRRC during the follow-ups, patients were recommended for a pelvic MRI and/or PET/CT. Every suspected case of LRRC was discussed in the Multidisciplinary Rectal Cancer Team meetings. Histologic verification of the recurrent disease was required, and tissue biopsies were conducted using CT guidance.

**Fig. 1:**
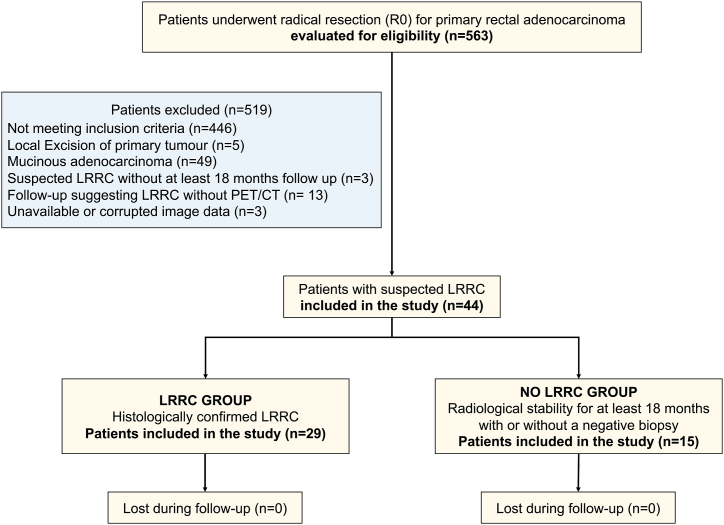
Flow chart of patient selection.

LRRC is defined as a recurrence of rectal cancer within the pelvis and after a prior surgical resection. This includes recurrence at the anastomotic site, tumor bed, or within lymphatic systems like residual mesorectal nodes and pelvic side-wall lymph nodes. Cases with positive biopsy results were classified under the LRRC group, while those showing no signs of LRRC in successive imaging tests over a period of at least 18 months, with or without a negative biopsy, were categorized as the NO LRRC group. A total of 29 LRRC cases (66 %) and 15 NO LRRC cases (34 %) were selected for the study, as shown in Fig. 1.

Patient demographics, tumor details and clinical characteristics were recorded in a dedicated database and explained in Table 1. All patients were monitored from the time of their primary tumor resection until their last check-up or death.

**Table 1:** Clinical characteristics of patients included in the study.

**Age (median, range)**		60 (33–84)
**Male gender**		61 % (27)
**Tumor distance from anal verge (median, range)**		5 cm (1–15)
**Neoadjuvant chemotherapy and/or radiotherapy**		79 % (39)
**Lymphovascular and/or perineural invasion**		48 % (21)
**Pathologic stage**	**pT1–2N0**	2 % (1)
	**pT3–4N0**	11 % (5)
	**pT1–4N+**	18 % (8)
	**ypT0N0**	9 % (4)
	**ypT1–2N0**	16 % (7)
	**ypT3–4N0**	9 % (4)
	**ypT1–4N+**	23 % (10)
	**T any N any M1**	2 % (1)
**Anastomotic leakage**		7 % (3)
**Adjuvant chemotherapy**		57 % (25)
**Time between primary tumor resection and LRRC diagnosis (median, range)**		13 months (2-77)

### 2.2 PET/CT image protocol acquisition

Patients underwent a standard 18F-FDG PET/CT. After fasting for at least 6 h, they received a single bolus injection of 18F-FDG, with the dose adjusted to range between 3.5 and 4.5 MBq/kg. Scans were taken 60 min post-injection (the uptake time), ensuring patients were hydrated with 500 mL of water and had previously emptied their bladder. Imaging was conducted using cross-calibrated GE Discovery MI, Discovery STE, or Discovery 710 devices (General Electric, Milwaukee, WI, USA), with each scan lasting 2 min per bed position. The scanning protocol adhered to the guidelines set by the European Association of Nuclear Medicine (EANM) [44].

### 2.3 Image segmentation and radiomic features extraction

The manual segmentation of the suspected rectal cancer recurrence was conducted on PET/CT studies. For the PET series, a nuclear medicine physician, P.C., with over two decades of experience in PET analysis, delineated the ROIs on the merged PET/CT images using Aliza Medical Imaging 1.98.18 (https://www.aliza-dicom-viewer.com/, accessed on November 30, 2024). Subsequently, the volume of interest (VOI) obtained was adjusted to match the original PET series resolution. Segmentation was executed in line with the guidelines co-established by the EANM and the Society of Nuclear Medicine and Molecular Imaging (SNMMI). An initial fixed threshold was set based on 40/50 % of the maximum Standardized Uptake Value (SUV). Adjustments and modifications were later made by the expert PET analyst due to the frequent occurrence of highly variable lesions within the study cohort [[45], [46], [47]].

**Fig. 2:**
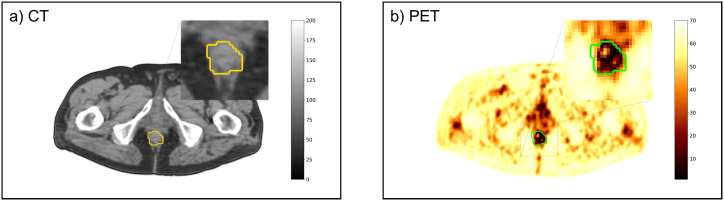
Manually segmented PET mask overlaid on CT (a) and PET (b) images.

The lesion VOIs segmented on PET images were translated into the CT images of the PET/CT study, as illustrated in Fig. 2, where a manually segmented PET mask is overlaid on the CT image (Fig. 2a) and the PET image (Fig. 2b). PET volume masks were used as input for a radiomic feature extraction pipeline, processing the CT and PET images independently.

Before radiomic feature extraction, PET images were corrected for the effect of 18F-FDG radionuclide decay. Each voxel was standardized according to the relation:

$${voxel}_{standardized}=voxel\;\cdot \;\frac{d\;\cdot \;e^{-\frac{t-t_{i}}{\tau }}}{w}$$

where *d* is radionuclide total dose, *t* is acquisition time, *t*_*i*_ is radiopharmaceutical start time, *τ* is radionuclide half-life and *w* is patient weight. No image interpolation was applied to preserve the integrity of voxel information.

From each lesion volume mask, a series of radiomic features from CT and PET images were extracted to evaluate characteristic descriptors of LRRC. At this stage, specific classes of radiomic features were not selected because it is challenging to know beforehand which features are most important. Instead, all features related to image intensity histogram, size and shape of pixel/voxel patterns, and voxel relationships were considered. In detail, the features extracted included *first-order statistics*, *3D shape-based scores*, *gray level co-occurrence matrix*, *gray level run length matrix*, *gray level size zone matrix*, *neighboring gray tone difference matrix* and gray level dependence matrix. Original images and transformed images obtained by applying the *Wavelet* (W) and *Laplacian of Gaussian* (LoG) transforms were considered for radiomic extraction. Thus, for each patient, a set of 1223 features were obtained from both CT and PET data. Radiomic feature extraction was performed using the pyRadiomics [48] python package.

### 2.4 Radiomic features selection and pipeline overview

The aim of the study was the prediction of LRRC through the analysis of radiomic features extracted from PET/CT images. In ML, this task can be treated as a binary classification problem in which the two classes are represented by healthy (NO LRRC) and recidivist (LRRC) patients. The radiomic pipeline developed consisted of three consecutive steps: i) extraction of radiomic features (in the range of thousands), ii) selection of a subset of radiomic features with the highest predictive power to eliminate redundant information, and iii) training of a supervised model for binary LRRC classification.

Following a standardization procedure, radiomics features were filtered using DNetPRO [41], a supervised network-based algorithm developed specifically for genomics data enabling the identification of a low dimensional set of features (a signature) for classification purposes. To the best of our knowledge, DNetPRO has never been used in a radiomic pipeline for feature selection, but it has proven suitable for study mainly because of two peculiarities: i) it can provide more easily interpretable results than other powerful methods, such as neural networks or SVMs; ii) its computational efficiency in filtering many features. Designing the DNetPRO algorithm requires setting up a classifier and a cross-validation scheme. Due to the limited sample size, in this study the evaluation of feature pairs and the identification of radiomic signatures were performed on the entire dataset with a 10-fold cross-validation using an SVC as classification model, ranking the features according to the Matthews's Correlation Coefficient (MCC), defined as:

$$MCC=\frac{TP\cdot TN-FP\cdot FN}{\sqrt{\left(TP+FP\;\right)\;\cdot \;\left(TP+FN\;\right)\;\cdot \;\left(TN+FP\;\right)\;\cdot \;\left(TN+FN\;\right)}}$$

where TP, TN, FP, and FN are the True Positive, True Negative, False Positive, and False Negative scores, respectively. The MCC, defined in the range [−1, +1], is a reliable metric for evaluating a binary classification that considers all categories of the confusion matrix (TP, TN, FP, FN). It is particularly suitable for the dataset used for the present study because it is robust to unbalanced classes. Segmentation performance was evaluated by using class balancing. Radiomic signatures were calculated independently from CT and PET features to evaluate their different predictive power.

The PET/CT signatures with the highest predictive scores were used to build an SVC with a non-linear (RBF) kernel. The classifier was evaluated in a K-fold cross-validation, allowing the training and testing in mutually exclusive folds, with K = 10. The choice K = 10, which is very common in the field of applied ML, was made as a good compromise between train-test splitting and sample numerosity, although there is no formal general rule [49,50]. In each fold a totally independent validation set was used to assess the predictive performance of the model, mimicking its application in a real clinical setting. The performance metrics of each fold were averaged out to estimate the overall goodness-of-fit of the model in predicting LRRC. The following metrics were used:

$$Sensitivity=\frac{TP}{TP+FN}$$

$$\text{Specificity}=\frac{TN}{TN+FP}$$

$$\text{BAS}=\frac{Sensitivity+Specificity}{2}$$

**Fig. 3:**
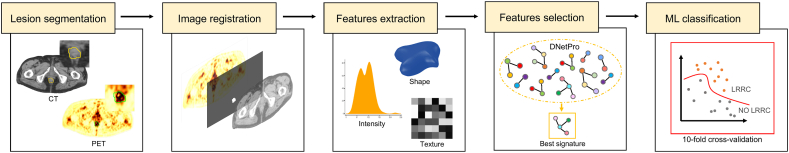
Outline of the proposed pipeline. For CT and PET images, after an image registration step, 1223 radiomic features were extracted from the segmented VOIs on PET images. Feature selection was performed on the entire radiomic dataset with the DNetPRO algorithm. To predict LRRC, an SVC model was trained in a 10-fold cross-validation with the best CT signature, PET signature and the combination of the best CT and PET signatures.

Sensitivity, specificity, and Balanced Accuracy Score (BAS) are standard metrics, defined in the range [0, 1], for evaluating the performance of a classification model. Sensitivity and specificity allow to assess how many positive and negative instances the model was able to correctly identify, respectively. BAS is an arithmetic mean of sensitivity and specificity and is designed to handle unbalanced datasets as in the present study. The best CT and PET signatures were combined to train an additional inclusive model to estimate the predictive capability of the combination of their features. All ML analyses were performed using the scikit-learn [51] python package. The scheme of the proposed pipeline is reported in Fig. 3.

## 3 Results

**Fig. 4:**
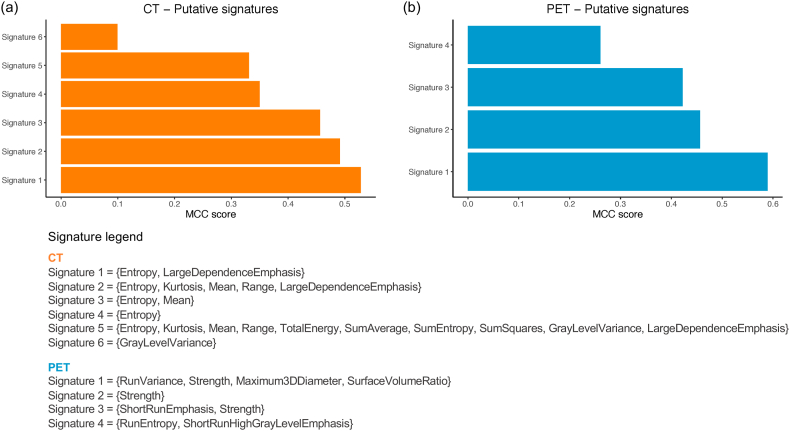
The MCC score of the putative signatures identified by the DNetPRO algorithm. For each signature, the radiomic features involved are listed in the legend. (a) MCC scores of the signatures selected from CT features. (b) MCC scores of the signatures selected from PET features.

**Fig. 5:**
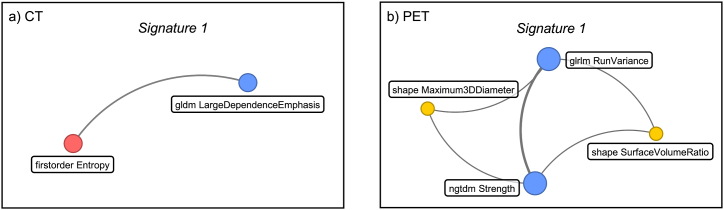
The network structure of the best signature, i.e. *Signature 1*, identified by the DNetPRO algorithm. Links between variables were estimated according to their effectiveness in classifying the two populations; more central nodes indicate the top player candidates for characterizing the two classes. For each node, the radiomic feature involved is specified in the label and its size is proportional to the quantity of in/out links. The node color is set according to the different type of radiomic quantity estimated: Haralick features (red), gray-level features (blue), and shape features (yellow). (a) *Signature 1* identified from the CT features. (b) *Signature 1* identified from PET features.

The radiomic pipeline was applied on the entire set of available volumes, extracting radiomic features on the manually identified lesion VOIs from CT and PET images independently. The MCC score of the radiomic signatures identified by DNetPRO from CT and PET features is shown in Fig. 4a and b, respectively. DNetPRO identified 6 different signatures for CT and 4 different signatures for PET datasets. The combination of radiomic features that make up each signature is reported in the legend inside Fig. 4. All features refer to the original image, i.e., the raw image without the application of any pre-processing filter. Transformed images (W and LoG filters) showed lower predictive powers. The SVC model was independently evaluated by considering for CT and PET the best signature identified by DNetPRO, i.e., *Signature 1*, whose network structure is shown in Fig. 5a and b, respectively. The results obtained with each evaluation metric by the selected CT, PET, and CT + PET features are reported in Table 2.

**Table 2:** Results of the SVC model trained in a 10-fold cross-validation with selected CT, PET, and CT + PET features.

Features	Sensitivity	Specificity	BAS	MCC
CT	0.80	**0.82**	**0.81**	**0.61**
PET	**0.93**	0.61	0.77	0.52
CT + PET	0.80	0.75	0.77	0.53

## 4 Discussion

Radiomics is an innovative and rapidly expanding field focused on the extraction and analysis of large amounts of quantitative features from medical images. When applied to oncology, this technique has the potential to unravel hidden patterns within imaging data that can predict the behavior of tumors and their recurrence. While conventional imaging provides a mostly qualitative view of tumors, radiomics can quantify tumor characteristics by extracting a large number (thousands) of features from standard-of-care images, such as CT, MRI, or PET images. The selection of the most predictive radiomic features from the thousands available is a crucial step in optimizing the final performance of the classification model. In this study, a new method for feature selection based on the use of DNetPRO is presented. The DNetPRO algorithm identified as best signatures subsets of variables made up from only 2 features for CT and 4 features for PET datasets, allowing a significant reduction in dimensionality that favors the interpretation of the radiomic signature from a radiological point of view. Furthermore, in the case of the PET dataset, the network description of the signature underscores the pivotal role of gray-level features compared with shape features, allowing an internal ranking of their informative power.

The SVC model with the selected CT radiomic signature yielded a sensitivity of 0.80, a specificity of 0.82, a BAS of 0.81 and an MCC of 0.61. Our findings are in line with current state-of-the-art evidence. Badic et al. [39] proposed three different ML models using CT images from two centers in combination with clinical variables, achieving as best result a BAS of 0.81 and an MCC of 0.61, with a required sensitivity of 95 %. Cuicchi et al. [40], proposed a single model trained and evaluated on 57 patients, achieving for the two selected CT radiomic features a sensitivity of 0.57 and 0.67 and a specificity of 1.00 and 0.71, respectively.

The SVC model with the selected PET radiomic signature performed worse, with a sensitivity of 0.93, a specificity of 0.61, a BAS of 0.77 and an MCC of 0.52. Such findings are still comparable with the reference literature, as in the aforementioned article by Cuicchi et al. [40], which obtained a sensitivity in the range of [0.71, 0.83] and a specificity in the range of [0.56, 0.63] with selected features from PET/CT imaging.

The results obtained from the CT + PET model (with a sensitivity of 0.80, specificity of 0.75, BAS of 0.77 and MCC of 0.53) showed that the combination of radiomic features selected from the two types of imaging brings no improvement in predictive performance. This suggests that the addition of selected PET features to selected CT features does not provide a significant advantage over using CT features alone. Additional evidence for this conclusion is provided by the application of the DNetPRO algorithm to the combined set of all CT and PET features. The algorithm identified the same best signature as when using only CT features, highlighting radiomic redundancy or lack of added predictive value from the PET modality. This finding seems to demonstrate that integrating CT and PET radiomic features does not enhance the performance compared to models based solely on CT features in this case, calling into question the value of incorporating PET data in this specific context.

The difference in SVC performance based on CT versus PET images showed the greater efficiency of CT features in capturing LRRC details. This may be due to the superior anatomical resolution of CT, which enables the identification of the heterogeneous texture of rectal lesions [52]. This hypothesis is supported by the signature selected by DNetPRO, which includes Entropy (a *first-order local feature* measuring the degree of randomness or complexity in the image intensity values within a VOI) and Large Dependence Emphasis (a *textural feature* measuring the distribution of large dependencies indicative of texture type). The signature, interpreted in terms of Hounsfield Units, corresponds to regions in the CT image characterized by significant inhomogeneity and distinct texture. Specifically, high Entropy values in CT images indicate significant tissue variability (often associated with tumor aggressiveness or complexity) and suggest regions containing mixed tissue types (such as necrotic or fibrotic areas, commonly observed in recurrent or advanced rectal cancers). Conversely, high Large Dependence Emphasis values denote regions of uniform intensity, often linked to fibrotic or scar tissue surrounding tumors. This may indicate a desmoplastic reaction, frequently observed in LRRC, and aid in identifying fibrotic scars that could obscure signs of recurrence.

In the case of PET images, DNetPRO selected 2 textural features, i.e., Run Variance, Strength, and 2 shape features, i.e., Maximum 3D Diameter, Surface Volume Ratio. The inspection of the signature network structure (shown in Fig. 5b) confirms the importance of textural features, including them as firmly bound central information that can cooperate with shape features. However, the predictive capability of the SVC model is likely hampered by the low resolution of PET images, which does not allow accurate quantitative analysis in each voxel as in the case of CT.

Understanding the role of imaging modalities in clinical diagnostics is crucial. Both CT and PET have their unique strengths: while PET is exceptionally sensitive and can detect metabolic changes often preceding anatomical alterations, CT is particularly valuable for its ability to provide a detailed anatomical visualization. Our findings highlight that the high spatial resolution and robust texture capabilities of CT make it a benchmark for the LRRC detection. However, it is important to note that in our pipeline, the extraction of radiomic features in CT images also benefited from functional information, since the VOIs of the lesions were manually segmented in PET images.

Our work indicates that radiomic features can be effectively used in the clinical setting to predict LRRC, a condition that remains difficult to manage. The ability of CT and PET features to detect subtle differences between noncancerous tissues and LRRC, which may not be easily distinguishable on conventional imaging, places radiomics as a valuable non-invasive tool for clinical practice. Integrating this radiomic model into routine diagnostic workflows could enhance early detection of LRRC, allowing for more precise monitoring and timely interventions.

The translational aspect of this study is particularly significant, as the proposed radiomics signature can be seamlessly integrated into clinical workflow to enhance diagnostic and therapeutic strategies. By automating feature extraction and analysis, the model provides actionable insights that enable clinicians to make informed decisions quickly and confidently. For example, the signature can stratify patients based on recurrence risk, guiding personalized follow-up protocols and targeted interventions. This capability may not only improve diagnostic precision but also optimize resource allocation, allowing for more focused and efficient patient management. Furthermore, its non-invasive nature reduces patient burden and supports the growing trend toward less invasive diagnostic paradigms.

It should be noted that the practical implementation of radiomics tools in clinical workflow brings challenges, such as ensuring standardized radiomic feature extraction and maintaining high image quality across different clinical settings. Despite these challenges, the potential benefits (improved diagnostic accuracy, reduced patient burden, and more personalized care) make radiomics a promising addition to the clinical toolkit for managing LRRC.

Our study had several limitations related to being based on a limited and unbalanced dataset, as patients were retrospectively recruited from a single medical center. Our sample size is relatively small from a statistical viewpoint, which may introduce biases and limit the generalizability of our results. However, it should be emphasized that, from a clinical perspective, this dataset represents a significant case series, as data were collected over an extended period at a large tertiary-level hospital, where patients are treated by renowned surgeons highly experienced in the field and disease recurrence rates are typically low. To address the challenges posed by the dataset size and imbalance, robust metrics specifically designed for unbalanced datasets were employed, such as the Balanced Accuracy Score (BAS) and Matthews Correlation Coefficient (MCC). Future research should focus on applying the proposed pipeline to a larger and diverse cohort of patients, ideally sourced from multiple medical centers. This would not only help validate the initial results but could also better account for the variability in patient demographics, treatment protocols, and clinical outcomes that a single-center study may fail to capture.

Despite the considerable clinical interest in this area, there is a notable paucity of articles in the existing literature. Most studies tend to focus on evaluating the response to neoadjuvant chemoradiotherapy, leaving a significant gap in understanding post-intervention outcomes. This gap underscores the novelty and importance of our study, which offers preliminary insights that could serve as a valuable foundation for future research. Expanding the dataset could substantially improve the application of these findings in clinical practice, potentially leading to more personalized and effective patient care. The key aspect of the study is its potential to reduce the reliance on invasive diagnostic procedures, such as biopsies. Currently, biopsies remain a common practice in the diagnostic process, particularly in complex cases. However, they are inherently invasive, carry associated risks, and can cause significant discomfort to patients. By advancing the methodologies explored in this study, there is a promising opportunity to develop non-invasive or minimally invasive diagnostic alternatives that could spare patients from the risks and burdens of biopsy, thereby improving overall patient experience and clinical outcomes. This direction not only aligns with the goals of precision medicine but also addresses a significant clinical need for safer and less invasive diagnostic tools. In addition, it would be interesting to evaluate the proposed pipeline with segmented VOIs in CT images to compare the predictive capabilities of CT and PET images in LRRC without any mutual influence.

## 5 Conclusions

The present study highlighted the importance of PET/CT radiomic features in predicting LRRC, which is still a challenging clinical problem. The selection of radiomic features with the highest predictive power was performed in an innovative way using DNetPRO, a supervised network-based algorithm. The original version of this algorithm was tailored to gene expression samples and has never been used in other biomedical contexts. The application of DNetPRO to the radiomic pipeline provided two main advantages: i) identification of small sets of the most informative features, i.e., the best signatures; ii) visualization of the relationships between selected features in a network structure for a finer grain description of the informative power of the involved variables and their statistical cooperation in the classification task. Both aspects facilitated the radiological interpretation of the PET/CT radiomic signature identified for LRRC classification. The results obtained from our pipeline can provide insights about the generalization capabilities of DNetPRO to other biomedical applications. The SVC model trained with CT putative signatures showed better LRRC identification ability than the models with PET putative signatures and combined CT + PET signatures. In particular, the radiomic features extracted from CT images (Entropy and Large Dependence Emphasis) proved effective in detecting the characteristic texture of LRRC.

Future work should focus on validating this model with larger, more diverse datasets to ensure its applicability across different patient populations and clinical settings. Additionally, exploring the integration of other imaging modalities, such as contrast-enhanced CT and MRI, could herald further improvements in predictive accuracy. Investigating the potential of multimodal approaches, combining radiomics with clinical and genetic data, could lead to even more comprehensive predictive models. Moreover, real-world clinical trials will be essential to assess the practical utility of these models in routine care and to refine them based on clinical feedback.

In summary, our proposed pipeline could provide a robust foundation for future use of radiomics and machine learning in the precision post-operative management of rectal cancer. With further research and validation, it has the potential to significantly transform the diagnostic landscape for LRRC.

## CRediT authorship contribution statement

**Sara Dalmonte:** Writing – original draft, Software, Methodology, Formal analysis. **Maria Adriana Cocozza:** Writing – original draft, Methodology, Data curation. **Dajana Cuicchi:** Writing – review & editing, Data curation. **Daniel Remondini:** Writing – review & editing, Supervision, Methodology. **Lorenzo Faggioni:** Writing – review & editing, Project administration. **Paolo Castellucci:** Writing – review & editing, Data curation, Conceptualization. **Andrea Farolfi:** Writing – review & editing, Data curation. **Emilia Fortunati:** Writing – review & editing, Data curation. **Alberta Cappelli:** Writing – review & editing, Investigation, Data curation. **Riccardo Biondi:** Writing – review & editing, Software, Methodology. **Arrigo Cattabriga:** Writing – review & editing, Data curation. **Gilberto Poggioli:** Writing – review & editing, Data curation. **Stefano Fanti:** Writing – review & editing, Data curation, Conceptualization. **Gastone Castellani:** Writing – review & editing, Supervision, Project administration. **Francesca Coppola:** Writing – review & editing, Data curation, Conceptualization. **Nico Curti:** Writing – original draft, Software, Methodology, Formal analysis.

## Informed consent statement

This study was an observational, retrospective single-center study and was approved by our local institution review board. Informed consent was waived by the institutional review board due to the retrospective nature of the study.

## Ethics statement

The study was conducted in accordance with the Declaration of Helsinki and was approved by the Institutional Review Board (or Ethics Committee) of IRCCS University Hospital of Bologna (protocol code no. 848/2020/OSS/AOUBo).

## Data and code availability

Data will be made available on request due to restrictions on privacy (European GDPR).

The code of DNetPro is available on Github at https://github.com/Nico-Curti/DNetPRO.

## Funding information

The study received no funding.

## Declaration of competing interest

The authors declare that they have no known competing financial interests or personal relationships that could have appeared to influence the work reported in this paper.
